# ACCESSIBLE, AFFORDABLE, AND SUSTAINABLE TRANSPORTATION INNOVATIONS FOR MOBILITY AND SAFETY AMONG OLDER ADULTS

**DOI:** 10.1093/geroni/igae098.1575

**Published:** 2024-12-31

**Authors:** Anu Siren, Judith Charlton

**Affiliations:** Chair; Discussant

## Abstract

While technology holds immense promise in addressing challenges related to safety, mobility, and sustainability in older adults’ transportation, significant barriers remain, particularly concerning the acceptability, affordability, and age-friendliness of these technologies. This symposium will feature presentations and discussions highlighting the multifaceted nature of mobility among older adults and the ways in which innovative technologies can address the transportation needs. The five presentations in this symposium communicate research from Australia, Europe, and the USA, and will explore issues related to usability, accessibility, and user acceptance, highlighting the importance of designing technologies that are intuitive, inclusive, and culturally sensitive. Speakers of this symposium will delve into the importance of mobility for older adults’ physical health, mental well-being, and social connectedness. Further, the symposium will examine the potential of technology-driven solutions to address key challenges faced by older adults in transportation, including safety concerns, accessibility barriers, and environmental sustainability. By fostering interdisciplinary dialogue and international collaboration, the symposium seeks to create multidimensional discussions that not only acknowledge the pivotal role of technology in addressing safety, mobility, and sustainability challenges for older adults but also address the issues of acceptability, affordability, and age friendliness. Transportation and Aging Interest Group Sponsored Symposium

